# Prognostic Value of Skull Base Foramen Invasion Subclassification in T Category Modification and Induction Chemotherapy Management for Nasopharyngeal Carcinoma: Post‐Hoc Analysis of a Dual‐Center Retrospective Cohort Study

**DOI:** 10.1002/advs.202408182

**Published:** 2024-12-04

**Authors:** Siyu Zhu, Shuqi Li, Di Cao, Chao Luo, Zhiying Liang, Shaobo Liang, Guoyi Zhang, Qin Zhao, Guangying Ruan, Lizhi Liu, Gui Fu, Haojiang Li

**Affiliations:** ^1^ Department of Radiology State Key Laboratory of Oncology in South China Guangdong Key Laboratory of Nasopharyngeal Carcinoma Diagnosis and Therapy Guangdong Provincial Clinical Research Center for Cancer Sun Yat‐sen University Cancer Center Guangzhou 510060 P. R. China; ^2^ Department of Radiation Oncology Foshan Academy of Medical Sciences Sun Yat‐Sen University Foshan Hospital and The First People's Hospital of Foshan Foshan 528000 P. R. China; ^3^ Department of Radiation Oncology The Third Affiliated Hospital of Sun Yat‐Sen University Guangzhou Guangdong 510000 P. R. China

**Keywords:** category modification, nasopharyngeal carcinoma, skull base foramen invasion, treatment management

## Abstract

Skull base foramen invasion (SBFI) indicates poor prognosis in nasopharyngeal carcinoma (NPC). However, only a few studies systematically assessed the role of SBFIin staging and treatment of NPC. To investigate the prognostic value of SBFI in NPC, a total of 1,752 patients with nonmetastatic NPC from two hospitals (1,320 and 432) between January 2010 and March 2014 are enrolled. The primary endpoint is overall survival (OS). Heatmap/cluster and network analyses are used to provide subclassification indication. Univariate and multivariate analyses with Kaplan–Meier method are performed to compare survival outcomes. SBFIs are classified into slight (only foramen lacerum and/or pterygopalatine fossa invasion) and severe (other SBFIs). The severe SBFI is an unfavorable prognosticator for OS in both the entire cohort and the T3 group. OS is similar between T3 with severe SBFI and T4 patients. Reclassifying T3 with severe SBFI as the T4 category yields an improved T category discrimination. Additionally, patients in the severe SBFI group gain significant survival benefits from induction chemotherapy ((IC). Therefore, T3 NPC with severe SBFI is an independent negative predictor for OS and is classified into the T4 category. T category adjustment enables better prognostic stratification. Severe SBFI benefits from IC in long‐term OS.

## Graphical abstract

A novel subclassification of skull base foramen invasion (SBFI) is established, based on its independent prognostic impact on nasopharyngeal carcinoma. This subclassification facilitates the identification of patients with severe SBFI, pinpointing who would benefit from induction chemotherapy. Furthermore, refining the T3 patients with severe SBFI into T4 enhances the risk discrimination of the T staging system.

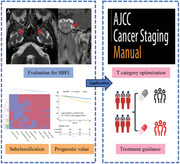

## 1 Introduction

Nasopharyngeal carcinoma (NPC) is a prevalent malignancy, particularly in Southeast Asia, with 133 354 newly diagnosed cases and 80 008 deaths reported worldwide in 2020.^[^ ^1^ ^]^ Clinically, NPC demonstrates an important growth pattern called “drilling” in which the tumor frequently invades the skull base foramen (SBF) and subsequently spreads to other structures through these channels.^[^ ^2^ ^]^ The foramina closely associated with NPC invasion include foramen lacerum (FL), foramen ovale, pterygopalatine fossa (PPF), jugular foramen, hypoglossal canal, superior orbital fissure, and inferior orbital fissure, which serve as pathways for the neurovascular structures to enter and exit the cranial cavity.^[^ ^3^ ^]^ Invasion of these foramina facilitates the spread of NPC to structures, such as the infratemporal fossa, orbit, and oropharynx.^[^ ^4^, ^5^, ^6^ ^]^ However, the current 8th edition of the American Joint Committee on Cancer (AJCC) staging system lacks specific descriptions of SBF involvement in T staging.

According to the AJCC 8th edition, skull base invasion (SBI) in NPC is classified as stage T3.^[^ ^7^ ^]^ Some studies have categorized the SBF invasion (SBFI) as part of SBI, considering them in the T3 category.^[^ ^4^, ^8^, ^9^ ^]^ However, other studies suggested the possibility of involvement of cranial nerves in patients with SBFI,^[^ ^5^ ^]^ such as cranial nerves IX–XI for jugular foramen^[^ ^10^ ^]^ and XII for hypoglossal canal,^[^ ^11^ ^]^ indicating destruction of distant structures observed in advanced stages of NPC (i.e., stage T4). These findings highlight the inadequacy of the current T staging system in incorporating SBFI.

Besides, previous studies attempted to optimize the staging of T3 patients according to the invasion of substructures because T3 category accounts for a large number of patients in the current staging system for whom the prognosis overlapped with T2 category.^[^ ^12^, ^13^, ^14^ ^]^ Considering the varied prognosis for patients with NPC having different SBFIs,^[^ ^8^, ^9^, ^15^ ^]^ the classification and identification of SBFI with similar prognosis are necessary to optimize current staging, improve prognosis prediction, and guide treatment decisions in patients with NPC.

Nowadays, patient prognosis in locoregionally advanced NPC remains unsatisfactory.^[^ ^16^ ^]^ The current recommended treatment for III–IVa NPC involves a combination of induction chemotherapy (IC) and concurrent chemoradiotherapy (CCRT) with Level 2A evidence.^[^ ^16^ ^]^ However, the treatment response to IC varies among patients.^[^ ^17^, ^18^ ^]^ Some studies have suggested that IC may not enhance survival in patients with T3‐4N0‐1 NPC.^[^ ^19^ ^]^ Additionally, IC brings increased toxicity of chemotherapy. Hence, it is crucial to identify patients who can derive benefits from IC, thereby mitigating the toxic side effects of chemotherapy for those who may not achieve similar benefits from IC. Heatmap/cluster and network analyses have been effective in analyzing complex biological datasets, thereby identifying clusters of highly correlated factors.^[^ ^20^, ^21^ ^]^ Our previous studies successfully used these methods to subclassify SBI^[^ ^14^ ^]^ and soft tissue involvement.^[^ ^22^ ^]^ In this study, we performed these analysis methods combined with classical statistics to subclassify SBFI and identify relationships between each SBFI site.

In this dual‐center retrospective study, the primary aim of this study is to evaluate the prognostic significance of SBFI in patients with NPC. After the post‐hoc analysis, we found a subclassification of SBFI in NPC based on survival outcomes. This finding leads to our secondary objectives, which were to determine whether the SBFI subclassification can be used to improve the T staging system and to validate its value in identifying potential candidates for IC.

## 2 Experimental Section

### 2.1 Study Design and Patients

This analysis was reported according to the Strengthening the Reporting of Observational Studies in Epidemiology recommendations.^[^ ^23^ ^]^ We performed a post hoc analysis of SBFI of patients with NPC. The ethical approval for this study was granted by the institutional ethics committees of the two hospitals (Approval No: B2019‐222‐01). Due to the retrospective nature of this investigation, informed consent was waived. The medical record review was performed for a total of 1752 consecutive patients who visited two different hospitals: Sun Yat‐sen University Cancer Center (Hospital 1, *n* = 1320) and the First People's Hospital of Foshan (Hospital 2, *n* = 432). Patient data from January 2010 to March 2014 (Figure S1A, Supporting Information) were retrospectively analyzed. The dual‐center datasets were polled into a single, unified database for further analysis to reduce the inter‐center bias. The sample size was estimated using the formulae for sample size and power in medical studies by M Woodward.^[^ ^24^ ^]^ We expected that the power should be over 95% with a type I error <5% for a two‐sided test. Through calculation, the minimum sample size requirement was 758 patients (295 patients with SBFI and 463 patients without SBFI). Our data size is larger than the minimal requirement. The inclusion criteria included the following factors: i) confirmation of NPC through pathological examination, ii) availability of complete pretreatment clinical information and laboratory data, iii) complete magnetic resonance imaging (MRI) data for the nasopharynx and neck regions, and iv) treatment with intensity‐modulated radiation therapy. The exclusion criteria were as follows: i) patients with insufficient medical records, including those with unknown follow‐up status or follow‐up time less than 3 months, and incomplete records with demographic characteristics, pretreatment plasma Epstein–Barr virus (EBV) DNA load, and treatment management, ii) patients with distant metastasis at first visit, iii) patients concurrently diagnosed with other types of tumors, and iv) patients with incomplete MRI findings for restaging assessment.

### 2.2 Follow‐up and Outcomes

During the 5‐year follow‐up period, the patients were scheduled for regular examinations at the hospitals, with appointments every 3 months during the initial 2 years and biannual appointments thereafter. Five‐year OS was regarded as the primary endpoint, calculating the time from the initial diagnosis to the date of death from any cause. Progression‐free survival (PFS) was calculated from the time from the initial diagnosis to the date of any disease progression, including distant metastasis, relapse, and death.

### 2.3 MRI Protocol

Detailed information on the MRI protocol is presented in the Supporting Information.

### 2.4 Imaging Assessment

**Figure 1:**
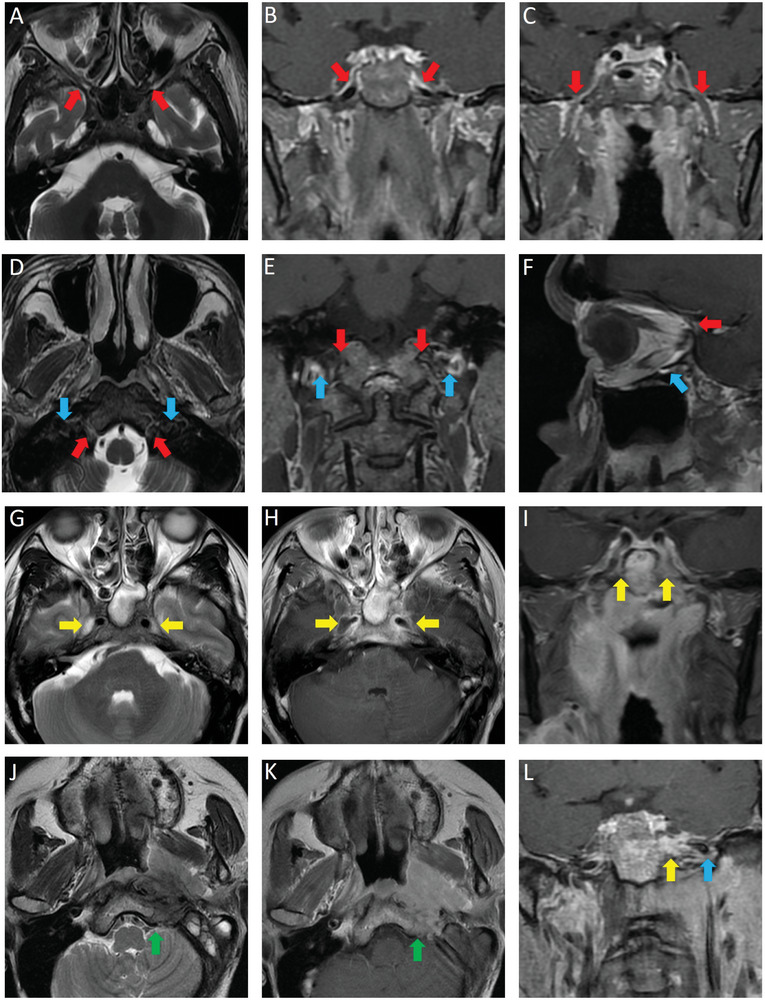
Diagram for the normal skull base foramen structures, representative MRI images illustrating slight SBFI and severe SBFI in patients with NPC. T2WI and contrast‐enhanced T1WI images illustrating normal skull base foramen position (A–F, arrows), including the pterygopalatine fossa (A), foramen lacerum (B), foramen ovale (C), hypoglossal canal (D, E, red arrow), jugular foramen (D, E, blue arrow), superior orbital fissure (F, red arrow), and inferior orbital fissure (F, blue arrow). Example of patients with NPC having slight SBFI (G–I): Patients with NPC with only bilateral foramen lacerum (yellow arrows) invaded were shown in T2WI (G), contrast‐enhanced T1WI (H), and coronal (I) images. Examples of patients with NPC having severe SBFI (J–L): left hypoglossal canal (J, K, green arrows), left foramen lacerum (L, yellow arrow), and foramen ovale (L, blue arrow) invaded were shown in T2WI (J), contrast‐enhanced T1WI (K), and coronal (L) images.

Two radiologists who specialized in head and neck cancers and had >10 years of clinical experience evaluated the MR images separately. The imaging finding for SBFI revealed widened SBFs, displaying mild‐to‐moderate brightness in T2‐weighted imaging replaced normal structural anatomy, and abnormal enhancement in contrast‐enhanced T1‐weighted imaging within the SBFs (**Figure** **1**). The optimal orientations for displaying each SBF were determined primarily based on the complete presentation of the SBF's shape and by comparing it with the opposite side by two readers. Any discrepancies were resolved through consensus. MRI assessment of SBFI in our study included the following sites: FL, foramen ovale, PPF, jugular foramen, hypoglossal canal, superior orbital fissure, and inferior orbital fissure.

### 2.5 Treatment

A detailed description of the treatment protocol is presented in the Supporting Information.

### 2.6 Heatmap/Cluster Analysis and Network Analysis

#### 2.6.1 Heatmap/Cluster Analysis

Using the clustering algorithm in the d3heatmap package within R software, heatmap/cluster analysis was performed. The cluster algorithm groups variables based on the similarity of their features, with the dendrogram visually presenting these grouping results. In our heatmap and cluster analysis, patients with any foramen invasion were selected and variables were foramen invasion. Each row represents the invasion status of different foramen in a single patient, and each column represents the invasion status of the same foramen across different patients. The dendrogram on the left of the clustering analysis results shows the clustering of patients based on the similarity of their foramen invasion characteristics, whereas the dendrogram on the top shows the clustering of different foramen invasions based on their distribution among patients.

To examine the correlation between different foramen invasions and prognosis, we innovatively incorporated primary endpoints—survival and death —into the heatmap and clustering analysis. “Survival” indicates patients who were still alive at the end of our follow‐up, whereas “dead” indicates patients who had died by the end of the follow‐up. Thus, in clustering, “close to survival” and “close to dead” indicate that, for foramen invasion, the patient's survival status is more associated with either survival or death according to the clustering grouping among variables.

#### 2.6.2 Network Analysis

Utilizing the “sna” package in R software, the network analysis employs graph theory to depict relationships between variables (nodes) and their interactions (edges). This approach utilizes various adjacency functions to convert co‐expression measures into connection weights, effectively illustrating classifications of different SBFIs through clustering. This method suggests potential subdivisions of variables into clusters formed by highly correlated factors. We have updated the code for this method in github: https://github.com/trackse/heatmap_network. This method has been successfully applied to the risk classification of soft tissue invasion among NPC.^[^ ^22^ ^]^

### 2.7 Statistical Analyses

The study flowchart is presented in Figure S1B (Supporting Information). To assess the consistency of SBFI assessments, kappa values were utilized within 184 patients with NPC. The chi‐squared test, Fisher's exact test, Student's *t*‐test, and Mann–Whitney U test were employed to compare distribution differences in baseline characteristics between slight SBFI and severe SBFI, as well as distribution differences in SBFI condition and baseline characteristics between the two hospitals. Heatmap/cluster and network analyses were further employed to classify patients with different SBFI structures according to the similarity in survival outcomes.

The Kaplan‐Meier method was employed to plot survival curves and estimate survival rates. The log‐rank test was used to compare survival differences in univariate analysis. Those variables related to OS (Table S1, Supporting Information) with *p* < 0.05 in univariate analysis were identified, and the confounding factors were finally selected based on multivariate Cox regression analysis with stepwise for confounding testing. Hazard ratios (HRs) with 95% confidence intervals (CIs) and the *p* value were calculated by multivariate Cox regression analysis with confounding factors. A forest plot was applied to visualize the HR (95% CI) for each SBFI structure and SBFI subclassification.

A 1:1 random matched‐pair analysis using confounding factors was conducted to compare the survival outcomes among III–IVa patients with different SBFI subclassifications treated with and without IC. The adjusted 5‐year prognosis was calculated for the proposed T category, considering the T3 subclassification. To evaluate the performance of the current and proposed T categories in survival prediction, the Harrell concordance index (C‐index) was used, with or without confounding factors, employing the U‐statistics test from the Hmisc package in R.

All statistical analyses mentioned above were conducted using R version 3.2.5 (https://www.r‐project.org/) and several packages, including stats, survival, rms, Hmisc, ggplot2, survminer, d3heatmap, ClustOfVar, network, sna, and GGally. Statistical significance was defined as a two‐tailed *p*‐value of <0.05.

### 2.8 Code Availability

The code for the sample size estimation and heatmap/cluster and network analysis has been uploaded to GitHub (https://github.com/trackse/dummy/blob/master/code_share_network_heatmap.r).

## 3 Results

### 3.1 Characteristics and Outcomes

The demographic and clinical characteristics of the patients at the two hospitals are shown in Table S1 (Supporting Information). The median follow‐up duration was 61.47 months. During follow‐up, 387 (22.09%) cases developed disease progress: 244 (13.93%) patients died, 225 (12.84%) patients developed distant metastases and 159 (9.08%) patients developed locoregional recurrence. As shown in Table S1 (Supporting Information), although the plasma EBV levels in the two hospitals were different, the staging of patients in the two hospitals was similar (*p* > 0.05). Univariate analysis demonstrated that age, sex, EBV, T category, N category, stage, and treatment were significant for OS (*p <* 0.05). After the confounding test, age, sex, T category, and N category were identified as confounding factors. EBV and treatment were eliminated after a stepwise procedure (Table S2, Supporting Information).

### 3.2 SBFI Subclassification

In the assessment of the reliability of SBFI, the kappa coefficients for inter‐reviewer identification were > 0.813 for the diagnosis of SBFI. In total, 682 of 1752 (38.9%) patients with NPC had SBFI. Of these, FL and PPF were the most common, with percentages of 27.60% (483/1752) and 21.6% (378/1752), followed by foramen ovale, hypoglossal canal, inferior orbital fissure, jugular foramen, and superior orbital fissure, respectively (**Table** **1**).

**Table 1:** Incidence and analysis of skull base foramen invasion for patients with NPC.

Variables	Total data	Hospital 1	Hospital 2		5‐year OS	
	[*n* = 1752]	[*n* = 1320]	[*n* = 432]	*p* value^a)^	Survival [%]	*p* value^b)^
Foramen lacerum				0.000		< 0.001
None	1269 (72.4%)	1016 (77.0%)	253 (58.6%)		88.71	
Yes	483 (27.6%)	304 (23.0%)	179 (41.4%)		76.24	
Pterygopalatine fossa				0.064		< 0.001
None	1374 (78.4%)	1049 (79.5%)	325 (75.2%)		87.74	
Yes	378 (21.6%)	271 (20.5%)	107 (24.8%)		76.12	
Foramen ovale				0.008		< 0.001
None	1439 (82.1%)	1066 (80.8%)	373 (86.3%)		87.41	
Yes	313 (17.9%)	254 (19.2%)	59 (13.7%)		74.92	
Hypoglossal canal				0.434		< 0.001
None	1593 (90.9%)	1196 (90.6%)	397 (91.9%)		86.49	
Yes	159 (9.1%)	124 (9.4%)	35 (8.1%)		72.79	
Jugular foramen				0.000		< 0.001
None	1684 (96.1%)	1283 (97.2%)	401 (92.8%)		86.38	
Yes	68 (3.9%)	37 (2.8%)	31 (7.2%)		59.15	
Superior orbital fissure				0.307		0.001
None	1740 (99.3%)	1309 (99.2%)	431 (99.8%)		85.5	
Yes	12 (0.7%)	11 (0.8%)	1 (0.2%)		48.89	
Inferior orbital fissure				0.004		0.021
None	1668 (95.2%)	1268 (96.1%)	400 (92.6%)		85.71	
Yes	84 (4.8%)	52 (3.9%)	32 (7.4%)		76.1	
SBFI 2 classification				0.202		< 0.001
Non‐severe	1352 (77.2%)	1009 (76.4%)	343 (79.4%)		88.49	
Severe	400 (22.8%)	311 (23.6%)	89 (20.6%)		73.86	
SBFI 3 classification				0.000		< 0.001
None	1070 (61.1%)	833 (63.1%)	237 (54.9%)		90.36	
Slight	282 (16.1%)	176 (13.3%)	106 (24.5%)		81.19	
Severe	400 (22.8%)	311 (23.6%)	89 (20.6%)		73.86	

**Abbreviations**: NPC, nasopharyngeal carcinoma; SBFI, skull base foramina invasion; none, patients without SBFI; slight SBFI, patients with invasion foramen lacerum and/or pterygopalatine fossa invasion only; non‐severe SBFI, patients without skull base foramina invasion and patients with only lacerum and/or pterygopalatine fossa invasion; severe SBFI, patients with other SBFIs

^a)^ *P* values were calculated for distribution differences between hospital 1 and hospital 2, using Fisher's exact test or the chi‐squared test for categorical variables; and

^b)^ *P* values were calculated by log‐rank test among the total cohort (*n* = 1752).

**Figure 2:**
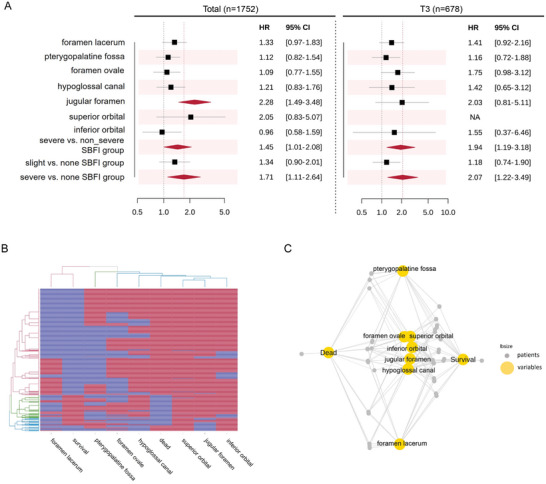
Multivariate analysis A), heatmap/cluster analyses B), and network analysis C) for patients with SBFI. In multivariate analysis (A), the jugular foramen is an independent prognostic factor for total patients, not for T3 patients; severe SBFI is an independent prognostic factor for both (A, red diamond, *p* < 0.05, black square, *p* ≥ 0.05). The heatmap/cluster analyses (B) show the clustering relationship can be categorized into the following: 1) foramen lacerum and survival, 2) pterygopalatine fossa, and 3) other SBFI and dead. Through network analysis (C), the SBFI was classified as follows: 1) foramen lacerum, 2) pterygopalatine fossa, 3) other SBFI, which is similar to the result from Figure 2B (Connections: Correlation between variables). **Abbreviations**: HR, hazard ratio; CI, confidence interval; SBFI, skull base foramen invasion; none, patients without SBFI; slight SBFI, patients with invasion foramen lacerum and/or pterygopalatine fossa invasion only; non_severe SBFI, patients without skull base foramina invasion and patients with only foramen lacerum and/or pterygopalatine fossa invasion; severe SBFI, patients with other SBFIs. **Note 1**. In Figure 2B (heatmap/cluster analyses), blue indicates the positive status of the variables, including the occurrence of foramen invasion, survival or death, and red indicates the negative status of the variables, including the absence of foramen invasion, dead, or survival. **Note 2**. Detailed result for multivariate analysis (Figure 2A) is presented in Table S4 (Supporting Information). **Note 3**. Given that patients with superior orbital fissure invasion are not categorized under the T3 stage, the application of HR for superior orbital fissure in this group is not applicable (NA).

The percentage of SBFI among 1752 patients with NPC within different T categories is shown in Table S3 (Supporting Information). Most patients with SBFI appeared in the T3 and T4 categories (Table S3, Supporting Information). Univariate analysis demonstrated that all SBFI sites were significant for OS (Table 1). After multivariate analysis with confounding factors, only jugular foramen was an independent prognostic factor for all patients (HR, 2.28; 95% CI, 1.49–3.48; *p* < 0.001) but not in T3 patients (HR, 2.03; 95% CI, 0.81–5.11; *p* = 0.131). Other SBF (skull base foramen) structures except jugular foramen were not independent OS predictors in the entire cohort or T3 subgroup (**Figure** **2**A; Table S4, Supporting Information).

Among patients with NPC with SBFI, the variables were classified into three groups according to clustering associations in heatmap/cluster analyses, including the following: 1) FL and survival, 2) PPF, and 3) other SBFI and dead (Figure 2B). The clustering classification demonstrated that FL is more associated with survival and that other SBFIs, except PPF, are more associated with death. Besides, network analysis classified the SBFI as follows: 1) FL, 2) PPF, and 3) other SBFIs (Figure 2C). As the single SBFI does not possess independent prognostic value, these provided evidence for the subsequent classification of SBFI variables.

### 3.3 Prognostic Value of SBFI for Patients with NPC

According to the aforementioned classification and further variables combination attempts, patients with NPC were classified into three SBFI subgroups: none (without SBFI), slight (only FL and/or PPF invasion), and severe (with others SBFI) SBFIs, with percentages of 61.1% (1070/1752), 16.1% (282/1752), and 22.8% (400/1752), respectively.

**Figure 3:**
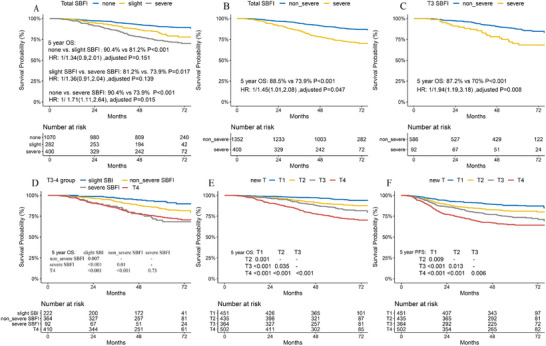
Prognostic stratification among patients with different SBFI classifications. Significant differences were observed between the none and severe SBFI groups in the 5‐year OS rate, whereas no significant difference was shown between the none and slight SBFI groups A). Combined with none and slight SBFI groups into a non‐severe group, severe SBFI demonstrated a poor 5‐year OS rate for all included patients B) and T3 patients C). Further classifying the T3 category with different SBI and SBFI subclassification, T3 patients with slight SBI, non_severe SBFI, and severe SBFI were discriminated significantly, but overlapped between T3 with severe SBFI and T4 groups D). Here, we suggested placing T3 patients with slight SBI into the T2 category, and T3 patients with severe SBFI into the T4 category. After this modification, a significant separation in prognosis for OS E) and PFS F) was observed in the proposed T category. **Abbreviations**: HR, hazard ratio; CI, confidence interval; OS, overall survival; PFS, progression‐free survival; SBFI, skull base foramen invasion; none, patients without SBFI; slight SBFI, patients with invasion foramen lacerum and/or pterygopalatine fossa invasion only; non_severe SBFI, patients without skull base foramina invasion and patients with only lacerum and/or pterygopalatine fossa invasion; severe SBFI, patients with other SBFIs; slight SBI, T3‐stage patients who skull base invasion only involved the pterygoid process and/or the base of sphenoid bone. **Note**. *p*‐value was calculated by log‐rank test; HR and adjusted *p*‐value were calculated by multivariable Cox regression with confounding factors.

Significant differences were observed between the none and severe SBFI groups with regard to the 5‐year OS rate (90.4% vs 73.9%; *p* < 0.001; HR: 1.71; 95% CI, 1.11–2.64; adjusted *p* = 0.015), whereas no significant difference was shown between the none and slight SBFI groups (90.4% vs 81.2%; *p* < 0.001; HR: 1.34; 95% CI, 0.90–2.01; adjusted *p* = 0.151) and between the slight SBFI and severe SBFI groups (81.2% vs 73.9%; *p* = 0.017; HR: 1.36; 95% CI, 0.91–2.04; adjusted *p* = 0.139) (**Figure** **3**A).

As the majority of patients with SBFI were in stages III–IVa (IVb patients were excluded from our study based on the exclusion criteria, due to the presence of distant metastasis), we conducted a comparison of the demographic and clinical characteristics among patients with slight and severe SBFI within this stratification (**Table** **2**) to identify potential factors contributing to the difference in OS. The findings revealed that patients in the severe SBFI group exhibited larger tumor volumes, elevated levels of EBV‐DNA, later T category, and overall stage compared with those in the slight group.

**Table 2:** Comparison of the characteristics of the groups based on SBFI among III‐IVa patients.

Variables	III‐IVa patients with SBFI	Slight	Severe	
	[n = 677]	[n = 278]	[n = 399]	*P* value^a)^
Age Median (IQR)	47(39‐56)	47 (40–56)	47 (38–56)	0.452
Sex				0.455
Male	519 (76.7%)	209 (75.2%)	310 (77.7%)	
Female	158 (23.3%)	69 (24.8%)	89 (22.3%)	
Histologic type^b)^				1.000
WHO type ½	14 (2.1%)	6 (2.2%)	8 (2.0%)	
WHO type 3	663 (97.9%)	272 (97.8%)	391 (98.0%)	
EBV (1 × 103 copies mL^−1^)				0.001
< 1	247 (36.5%)	122 (43.9%)	125 (31.3%)	
< 10	222 (32.8%)	89 (32%)	133 (33.3%)	
≥ 10	208 (30.7%)	67 (24.1%)	141 (35.3%)	
T category^c)^				<0.001
T3	294 (43.4%)	202 (72.7%)	92 (23.1%)	
T4	383 (56.6%)	76 (27.3%)	307 (76.9%)	
N category^c)^				0.106
N0	89 (13.1%)	40 (14.4%)	49 (12.3%)	
N1	403 (59.5%)	168 (60.4%)	235 (58.9%)	
N2	143 (21.1%)	48 (17.3%)	95 (23.8%)	
N3	42 (6.2%)	22 (7.9%)	20 (5%)	
Stage^c)^				<0.001
III	272 (40.2%)	184 (66.2%)	88 (22.1%)	
Iva	405 (59.8%)	94 (33.8%)	311 (77.9%)	
GTV (cm^3^) Median (IQR)	52.7 (34.1‐80.5)	39.8 (26.1–58.9)	64.9 (42.2–90)	<0.001
lymph nodal number Median (IQR)	3(1‐5)	3 (1–5)	3 (1–6)	0.087

Abbreviation: SBFI, skull base foramina invasion; Slight, patients with only lacerum and/or pterygopalatine fossa invasion; Severe, patients with other SBFIs; EBV, Epstein–Barr virus; IQR, interquartile range; WHO, World Health Organization; GTV, gross tumor volume.

^a)^ *p‐*values calculated using the chi‐squared test or Fisher's exact test for categorical variables and Student’s t‐test or Mann‐Whitney U‐test for continuous variables;

^b)^ According to the 2005 World Health Organization classification of tumors; and

^c)^ According to the 8th edition of the American Joint Committee on Cancer staging system.

Based on the results from heatmap/network analysis and multivariate analysis, we combined none and slight SBFI groups as non_severe groups. Compared with the severe SBFI group, the non_severe SBFI showed better 5‐year OS among all patients (88.5% vs 73.9%; *p* < 0.01; HR: 1.45; 95% CI, 1.01–2.08; adjusted *p* = 0.047) and T3 patients (87.2% vs 70.0%; *p <* 0.001; HR: 1.94; 95% CI, 1.19–3.18; adjusted *p* = 0.008) (Figures 3B,C and 2A and Table S4, Supporting Information). The adverse prognostic value of severe SBFI among T3 patients was validated across each institution (Figure S2, Supporting Information).

In our previous study regarding SBI using the same patient database, we found that slight SBI (T3 patients with pterygoid process and/or base of the sphenoid bone invasion only) was recommended to downstage to T2 category because of higher OS and PFS rates than those of severe SBI (T3 patients with other SBIs).^[^ ^25^ ^]^ In this study, we considered the subclassification of SBI; severe SBIs were further subclassified according to the extent of SBFI. Finally, T3 patients were grouped as those with slight SBI, non_severe SBFI, and severe SBFI. Patients grouped as those having slight SBI, non_severe SBFI, and severe SBFI (92.7% versus 83.8%, *p* = 0.007; 83.8% vs 70.0%, *p* = 0.01; 92.7% vs 70.0%, *p*<0.001) showed significant differences in the 5‐year OS. However, no significant difference was noted between the severe SBFI and T4 groups (70% vs 74.1%, *p* = 0.73) (Figure 3D). Besides, the 5‐year OS overlapped among T4 patients with different SBFI subclassifications. T3 patients with non_severe SBFI presented with a better OS compared with the rest of the patients among the T3–4 groups (*p* < 0.05) (Figure S4, Supporting Information). Thus, we proposed reclassifying T3 category patients with severe SBFI as the new T4 category and T3 patients with slight SBI as the T2 category. According to the 8th AJCC staging system, the survival curve of T2 category patients almost overlapped with that of T3 patients.^[^ ^14^, ^25^ ^]^ In our cohort, the OS for T2 and T3 categories almost overlapped in both hospitals (Figure S3, Supporting Information). After this revision, a significantly separated prognosis for OS (Figure 3E) and PFS (Figure 3F) was observed in the proposed T category in the total cohort (all *p* < 0.05). The survival outcomes for PFS among SBFI subclassification in T3 and T4 patients were consistent with the OS results (Figure S5, Supporting Information). The C‐index of the proposed T category in OS prediction improved but was not significant compared with the 8th edition T staging system across both the train and test cohorts whether considering the T category alone or considering the T category and confounding factors together (Table S6, Supporting Information).

### 3.4 Treatment Outcomes for IC

**Figure 4:**
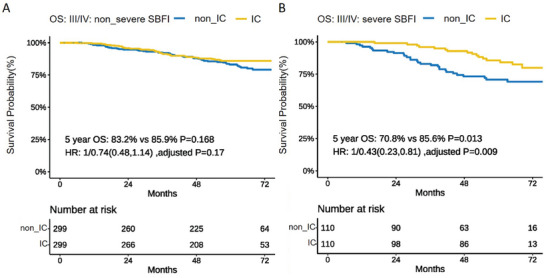
Survival outcomes for 5‐year OS among SBFI subclassification treated with or without induction chemotherapy. Among patients with stage III–IV NPC, patients with non‐severe SBFIdid not gain significant survival benefits for 5‐year OS from additional IC A); significantly improved 5‐year OS was observed in patients with severe SBFI treated with additional IC than in those without IC B). **Abbreviations**: HR, hazard ratio; IC, induction chemotherapy; non_IC, without IC treatment; OS, overall survival; SBFI, skull base foramen invasion; non_severe SBFI, patients without skull base foramina invasion and patients with only lacerum and/or pterygopalatine fossa invasion; severe SBFI, patients with other SBFIs. **Note 1**. A 1:1 random matched‐pair analysis using confounding factors were conducted. *P* value was calculated by log rank test; HR and adjusted P value were calculated by multivariate Cox regression with confounding factors. **Note 2**. Basic characteristics for the pairs of with severe SBFI and non_severe SBFI group are presented in Table S5 (Supporting Information).

A total of 299 pairs of patients with non_severe SBFI and 110 pairs with severe SBFI for patients treated with or without IC were carefully selected in 1:1 random matched‐pair analysis and matched by the confounding factors of T category, N category, age, and sex among III–IVa patients with SBFI subclassification. Detailed information regarding the basic characteristics is presented in Table S5 (Supporting Information). In the non_severe SBFI group, no significant differences were observed in 5‐year OS between patients treated with or without IC (83.2% vs 85.9%; *p* = 0.168; HR: 0.74; 95% CI, 0.48–1.14; adjusted *p* = 0.17). In contrast, in the severe SBFI group, patients who received IC exhibited a significant survival benefit in terms of 5‐year OS, compared with those without IC (70.8% vs 85.6%; *p* = 0.013; HR: 0.43; 95% CI, 0.23–0.81; adjusted *p* = 0.009) (**Figure** **4**).

## 4 Discussion

In our dual‐center study, we retrospectively investigated the prognostic value of SBFI in patients with NPC. We categorized SBFI into two grades, slight (only FL and/or PPF invasion) and severe (other SBFs, including foramen ovale, jugular foramen, hypoglossal canal, superior orbital fissure, and inferior orbital fissure invasion), based on the difference in the 5‐year OS rate. Severe SBFI was an independent negative prognostic factor for adverse OS in both patients with NPC and those with T3 stage. Additionally, among III–IVa patients with SBFI, we found that patients with severe SBFI could gain benefit from IC in long‐term OS, rather than patients with non_severe SBFI. Considering the previous proposal of T3 patients with different SBIs, we subclassified T3 patients as slight SBI (pterygoid process and/or base of the sphenoid bone invasion only), non_severe SBFI (none and slight SBFI), and severe SBFI according to their condition of SBI and SBFI. It is found that classifying T3 patients with slight SBI into T2 category, as reported in our last study,^[^ ^25^ ^]^ T3 patients with severe SBFI classified into the T4 category yielded better discrimination in T category.

Our previous studies focused on subclassifying SBI to guide prognostic stratification and IC usage,^[^ ^14^, ^25^ ^]^ but failed to consider the prognostic value of SBFIs in NPC. Some previous studies combined SBI and SBFI together into the SBF grading system,^[^ ^4^, ^8^, ^9^ ^]^ but did not analyze the survival difference of each foramen invasion separately, leaving the independence of these factors uncertain. Additionally, grading criteria based on invasion percentage favored classifying SBFI as severe, as it typically has a lower percentage than SBI. Thus, in our study, we performed univariate and multivariate analyses for each foramen invasion. The results showed that the invasion of jugular foramen was the only independent prognostic factor for OS in the entire study population but not T3 stage patients; however, the percentage of jugular foramen invasion was relatively low (68/1752). Other foramina were not significant independent factors for OS both in the entire study population and patients with the T3 stage of NPC, leading us to explore potential prognostic subgroups. The heatmap/cluster and network analyses proposed the subclassification of SBFI into three groups with clustering associations: (1) FL, (2) PPF, and (3) other SBFI, providing evidence for further SBFI subclassification in prognosis. The further survival analysis validated that the invasion with only FL and/or PPF was more associated with survival, whereas the other foramina invasion clustered together and was more associated with death, demonstrating an independent prognostic value of SBFI subclassification.

Patients with FL and PPF invasion (slight SBFI group) had a more favorable prognosis than those with other SBFIs. Their prognosis was similar to that of patients without SBFI. The 8th edition of the AJCC update introduced a description of pterygoid structures in the T3 staging, encompassing the medial plate/lateral plate, base of the pterygoid process, maxillary fissure of the pterygoid process, and the PPF.^[^ ^7^ ^]^ The inclusion of PPF as part of the pterygoid structures in T3 staging supports our findings that PPF invasion is classified as slight SBFI, which is associated with a more favorable prognosis. Moreover, a recent study revealed that 78% of patients with PPF invasion had FL invasion through the vidian canal, making it one of the most common invasion routes in PPF‐invaded patients.^[^ ^26^ ^]^ Additionally, Cao et al. reported that among patients with FL invasion, the rate of PPF invasion reached 39.2%, which represented an approximate 10% increase, compared with PPF invasion in all patients.^[^ ^27^ ^]^ These studies suggest a certain correlation between the occurrence of these two SBFs, further supporting the rationality for categorizing them into one classification.

The positions of these two skull base foramina are typically closer to the midline, adjacent to the primary tumor, unlike the more eccentric locations of other foramina (Figure 1). This proximity makes them susceptible to infiltration even when the primary tumor volume is small, which explains the higher percentage than in other SBFIs. These data are consistent with those of previous studies.^[^ ^8^, ^26^ ^]^ We observed that the slight SBFI group had a smaller gross tumor volume than the severe group (39.8 cm^3^ versus 64.9 cm^3^, *p <* 0.001) (Table 2), which aligns with previous findings that tumor volume significantly affects the prognosis of patients with NPC.^[^ ^28^ ^]^

In the severe SBFI group, patients with invasion of foramen ovale, inferior orbital fissure, superior orbital fissure, jugular foramen, and hypoglossal canal had a worse prognosis. These foramina are located eccentrically and with a larger tumor volume. Moreover, the presence of nerves within these channels increases the likelihood of invading T4 structures, resulting in a poorer prognosis. Invasion of structures, such as the cavernous sinus posterior to the foramen ovale,^[^ ^3^, ^7^ ^]^ involvement of the orbital fissures affecting the orbit,^[^ ^4^ ^]^ and extension into the intracranial region through the jugular foramen and hypoglossal canal are examples.^[^ ^10^, ^29^ ^]^ Metastasis to these structures contributes to poorer survival rates when these foramina are involved.

The severe SBFI group exhibited significantly larger tumor volumes and higher levels of EBV‐DNA, compared with the slight group. Additionally, the severe group had a later T category and overall stage, indicating a higher tumor burden in the severe SBFI group. However, there were no significant differences in N staging and lymph nodal number between the two groups, suggesting a limited impact on lymph node‐related factors in the severe group. Nasopharyngeal carcinoma, associated with EBV, shows a correlation between higher EBV DNA load and advanced stages.^[^ ^30^ ^]^ Elevated EBV DNA levels are associated with adverse prognostic factors, such as metastasis and recurrence.^[^ ^31^, ^32^ ^]^ Our results align with these findings, showing that patients in the severe group exhibit higher EBV levels, later staging, and poorer prognosis.

The current AJCC staging system has an excessive number of T3 cases with significant prognostic differences, which is consistent with our T stage distribution,^[^ ^12^, ^13^, ^14^ ^]^ suggesting a need for T3 subclassification in the staging system to improve prognosis prediction for patients with T3 category NPC. Based on the research from SBI subclassification,^[^ ^14^, ^25^ ^]^ we further considered the prognostic role of SBFI. In the proposed T3 subclassification, significant survival analysis was observed among them. Moreover, a similar OS rate was shown between severe SBFI and T4 category, suggesting that severe SBFI classified as T4 category was reasonable. Meanwhile, as we reported in the previous study about SBI, slight SBI was recommended to downstage to the T2 category. After this staging adjustment, the revised T category showed better prognosis discrimination for OS and PFS, compared with the current AJCC system. This refinement can help enhance the accurate risk stratification in the T stage for patients with NPC and guide treatment strategies.

The current recommended treatment for patients with advanced NPC involves a combination of IC and CCRT with Level 2A evidence.^[^ ^16^ ^]^ Although IC improves survival outcomes in locally advanced NPC, it is also accompanied by increased toxicity.^[^ ^17^, ^18^, ^33^, ^34^ ^]^ Moreover, there is variability in treatment response to IC, and not all patients can benefit equally despite with the same stage of locally advanced disease.^[^ ^17^, ^18^, ^33^, ^34^ ^]^ Some studies have suggested that IC may not improve survival in patients with T3‐4N0‐1 NPC.^[^ ^19^ ^]^ In our study, we observed that patients with III–IVa stage disease with severe SBFI could gain benefit from IC in long‐term OS. These patients often have a larger tumor burden, and IC can help reduce it. Conversely, patients with slight SBFI have a smaller tumor volume, and the position is closer to the primary tumor. Radiotherapy could achieve a satisfactory therapeutic effect. The grading system could help patients with slight SBFI avoid unnecessary treatment and associated toxicity and receive more moderate therapeutic management. Our SBFI grading system enables more appropriate therapeutic approaches, leading to better stratification and improved survival outcomes for patients with NPC.

### 4.1 Limitations

First, this study was limited by the small size of the skull base foramina and the lack of clear invasion criteria. Thus, we performed analysis strictly controlling the MRI evaluation standards to ensure the accuracy of clinical judgments on NPC invasion by different doctors. Second, because surgery is not the definitive treatment for NPC, pathological evidence of foramina invasion is unattainable because of the distinct anatomical position. The good kappa value indicated the minimization of evaluation mistakes. Finally, this was a dual‐center post hoc analysis with a relatively small population in the second hospital. Larger sample sizes, multicenter studies, and prospective studies are required to fully assess the prognostic value of SBFI subclassification in T category modification and IC management.

## 5 Conclusion

Our newly proposed subclassification of severe SBFI was an independent adverse prognostic factor for OS in patients with NPC. SBFI grading system could further subclassify T3 patients to achieve a more satisfactory risk stratification and might be used to optimize T staging. Patients with severe SBFI might benefit from IC in long‐term OS.

## Conflict of Interest

The authors declare no conflict of interest.

## Author Contributions

S.Z., S.L., D.C., and C.L. contributed equally to this study. S.Z., G.F., H.L., and L.L. conceived and designed the study. S.Z., S.L., D.C., C.L., G.F., and H.L. designed and performed experiments, analyzed data, and wrote the manuscript. S.L., D.C., Z.L., G.F., S.L., G.Z., and G.R. supervised the study and provided data. All authors wrote, reviewed, or edited the manuscript.

## Supporting information



Supporting Information

## Data Availability

The data that support the findings of this study are available from the corresponding author upon reasonable request.
