# Supplementary material for: Prognostic Value of Skull Base Foramen Invasion Subclassification in T Category Modification and Induction Chemotherapy Management for Nasopharyngeal Carcinoma: Post‐Hoc Analysis of a Dual‐Center Retrospective Cohort Study
Source: Adv Sci (Weinh). 2024 Dec 4;12(4):2408182. doi: 10.1002/advs.202408182 (PMC11789575; doi:10.1002/advs.202408182)
Supplement: Supplementary file 1 — Supporting Information [file ADVS-12-2408182-s001.docx]

**Supporting Information**

**for**

**Prognostic Value of Skull Base Foramen Invasion Subclassification in T Category Modification and Induction Chemotherapy Management for Nasopharyngeal Carcinoma: Post-hoc Analysis of a Dual-center Retrospective Cohort Study**

*Siyu Zhu, Shuqi Li, Di Cao, Chao Luo, Zhiying Liang, Shaobo Liang, GuoYi Zhang, Qin Zhao, Guangying Ruan, Lizhi Liu*, Gui Fu*, Haojiang Li**

1. **Supplementary methods**

MRI protocol

Treatment

1. **Supplementary tables**

Table S1. Demographic and clinical characteristics of the participants

Table S2. Procedures of confounding testing by multivariable Cox regression related to overall survival.

Table S3. Percentage of skull base foramen invasion with different T categories (N=1752)

Table S4. Multivariable analysis of skull base foramen invasion for total and T3 NPC patients

Table S5. Basic characteristics of SBFI subclassification treated with or without induction chemotherapy in 1:1 random matched-pair analysis.

Table S6 C-index of the proposed T staging system in NPC patients

1. **Supplementary figures**

Figure S1. Flowchart of the study: patient recruitment (A) and procedure of analysis (B).

Figure S2. Independent validation of prognostic value of severe SBFI in T3 patients with nasopharyngeal carcinoma.

Figure S3. The 5-year OS for T staging according to the 8th edition AJCC system for hospital 1 and 2.

Figure S4. Survival outcomes for 5-year OS among SBFI subclassification in T3 and T4 patients with nasopharyngeal carcinoma.

Figure S5. Survival outcomes for PFS among SBFI subclassification in T3 and T4 patients with nasopharyngeal carcinoma.

**Supplementary methods**

**MRI protocol**

Pretreatment MRI examination was performed using a 1.5-T system (Signa CV/i, General Electric Healthcare) or a 3.0-T system (Magnetom Tim Trio, Siemens). The scanning range, with a combined head and neck coil, ranged from the suprasellar cistern to the inferior margin at the sternal end of the clavicle. Unenhanced fast-spin echo (FSE) T1-weighted imaging (T1WI) in the axial, coronal, and sagittal planes (repetition time [TR]=540 ms, echo time [TE]=11.8 ms), unenhanced FSE T2-weighted imaging in the axial plane (TR=4000 ms, TE=99 ms), contrast-enhanced (CE) T1WI in the axial and sagittal planes and fat-suppressed CE T1WI imaging in the coronal plane were performed for all patients. A contrast agent (Gd-DTPA [0.01 mmol/kg]; Magnevist, Schering) was injected intravenously. The FSE sequences using a scan plane of oblique, flip angle of 80°, number of excitations of 4, field of view of 24cm, and matrix of 24×24. The section thicknesses were 5 mm, 3 mm, and 2 mm for the axial, sagittal, and coronal planes, respectively. The section gaps were 1 mm for the axial and sagittal planes, and 0.5 mm for the coronal plane.

**Treatment**

In IMRT, 68 Gy and 60–64 Gy were delivered to the planning target volume of primary gross tumor volume and the involved lymph nodes. Clinical target volumes with high-risk, low-risk, and neck nodal regions received 60 Gy, 54 Gy, and 54 Gy, respectively. The radiation schedules were 1 fraction per day for 5 days per week. The IMRT period was 6–7 weeks. Chemotherapy regimens for CCRT were based on cisplatin, with 30–40 mg/m^2^ per week or 80–100 mg/m^2^ for 2–3 cycles in 3 weeks. IC was delivered before IMRT for 2–3 cycles within 21 days, with 80 mg/m^2^ cisplatin plus 1000 mg/m^2^ 5-fluorouracil, 75 mg/m^2^ cisplatin plus 75 mg/m^2^ docetaxel, or 60 mg/m^2^ cisplatin plus 600 mg/m^2^ 5-fluorouracil plus 60 mg/m2 docetaxel.

**Supplementary tables**

**Table S1.** Demographic and clinical characteristics of the participants

| Variables | Total data | Hospital 1 | Hospital 2 |  | 5-year OS | |
| --- | --- | --- | --- | --- | --- | --- |
|  | (*N* = 1752) | (*N* = 1320) | (*N* = 432) | *P*_1_value | Survival (%) | *P*_2_ value |
| Age  Median (IQR) | 46 (39-55) | 45 (38–54) | 47 (40–58) | <0.001 | - | <0.001 |
| Sex |  |  |  | 0.306 |  | 0.006 |
| Male | 1297 (74.0%) | 969 (73.4%) | 328 (75.9%) |  | 83.95 |  |
| Female | 455 (26.0%) | 351 (26.6%) | 104 (24.1%) |  | 88.99 |  |
| Histologic type^※^ |  |  |  | <0.001 |  | 0.269 |
| WHO type 1/2 | 63 (3.6%) | 63 (4.8%) | 0 (0.0%) |  | 79.40 |  |
| WHO type 3 | 1689 (96.4%) | 1257 (95.2%) | 432 (100.0%) |  | 85.49 |  |
| EBV |  |  |  | <0.001 |  | <0.001 |
| (1 × 10^3^ copies/mL) |  |  |  |  |  |  |
| <1 | 852 (48.6%) | 583 (44.2%) | 269 (62.3%) |  | 90.13 |  |
| <10 | 493 (28.1%) | 339 (25.7%) | 154 (35.6%) |  | 79.56 |  |
| ≥10 | 407 (23.2%) | 398 (30.2%) | 9 (2.1%) |  | 81.62 |  |
| T category‡ |  |  |  | 0.104 |  | <0.001 |
| T1 | 451 (25.7%) | 331 (25.1%) | 120 (27.8%) |  | 95.16 |  |
| T2 | 213 (12.2%) | 153 (11.6%) | 60 (13.9%) |  | 85.45 |  |
| T3 | 678 (38.7%) | 532 (40.3%) | 146 (33.8%) |  | 85.16 |  |
| T4 | 410 (23.4%) | 304 (23%) | 106 (24.5%) |  | 74.08 |  |
| N category‡ |  |  |  | 0.099 |  | <0.001 |
| N0 | 352 (20.1%) | 278 (21.1%) | 74 (17.1%) |  | 93.05 |  |
| N1 | 993 (56.7%) | 752 (57%) | 241 (55.8%) |  | 87.37 |  |
| N2 | 289 (16.5%) | 205 (15.5%) | 84 (19.4%) |  | 75.68 |  |
| N3 | 118 (6.7%) | 85 (6.4%) | 33 (7.6%) |  | 65.59 |  |
| Stage |  |  |  | 0.409 |  | <0.001 |
| I | 151 (8.6%) | 115 (8.7%) | 36 (8.3%) |  | 97.49 |  |
| II | 388 (22.1%) | 288 (21.8%) | 100 (23.1%) |  | 94.24 |  |
| III | 708 (40.4%) | 547 (41.4%) | 161 (37.3%) |  | 85.83 |  |
| IV | 505 (28.8%) | 370 (28%) | 135 (31.2%) |  | 73.33 |  |
| Treatment |  |  |  | 0.004 |  | 0.013 |
| IMRT | 225 (12.8%) | 156 (11.8%) | 69 (16.0%) |  | 89.23 |  |
| CCRT | 635 (36.2%) | 504 (38.2%) | 131 (30.3%) |  | 87.32 |  |
| IC+CCRT | 892 (50.9%) | 660 (50%) | 232 (53.7%) |  | 82.72 |  |

**Abbreviations:** OS, overall survival; EBV, Epstein–Barr virus; WHO, World Health Organization; CCRT, concurrent chemoradiotherapy; IMRT, intensity-modulated radiation therapy; IC, induction chemotherapy

* *P*_1_ values were calculated for distribution differences between hospital 1 and hospital 2 using Fisher’s exact test or the chi-squared test for categorical variables, and student t test for continuous variables. *P*_2_ value was calculated by log-rank test among total cohort (n=1752).

^※^According to the 2005 World Health Organization classification of tumors.

^‡^According to the 8th edition of the AJCC staging system.

**Table S2**. Procedures of confounding testing by multivariable Cox regression related to overall survival.

| Variables |  |  | Without stepwise | |  | With stepwise | |
| --- | --- | --- | --- | --- | --- | --- | --- |
|  |  |  | HR (95%CI) | *P* value |  | HR (95%CI) | *P* value |
| T category |  |  |  |  |  |  |  |
| T1 | 451(25.7%) | | 1(reference) | |  | 1(reference) | |
| T2 | 213(12.2%) | | 2.3(1.31-4.05) | 0.004 |  | 2.29(1.30-4.02) | 0.004 |
| T3 | 678(38.7%) | | 2.64(1.64-4.23) | <0.001 |  | 2.57(1.61-4.1) | <0.001 |
| T4 | 410(23.4%) | | 5.1(3.14-8.27) | <0.001 |  | 4.79(3.01-7.64) | <0.001 |
| N category |  |  |  |  |  |  |  |
| N0 | 352(20.1%) | | 1(reference) | |  | 1(reference) | |
| N1 | 993(56.7%) | | 1.67(1.06-2.63) | 0.026 |  | 1.59(1.02-2.47) | 0.040 |
| N2 | 289(16.5%) | | 3.24(1.97-5.32) | <0.001 |  | 3.05(1.90-4.89) | <0.001 |
| N3 | 118(6.7%) |  | 5.87(3.33-10.35) | <0.001 |  | 5.27(3.14-8.84) | <0.001 |
| Age | 1752(100%) | | 1.03(1.02-1.04) | <0.001 |  | 1.03(1.02-1.04) | <0.001 |
| Sex |  |  |  |  |  |  |  |
| Male | 1297(74.0%) | | 1(reference) | |  | 1(reference) | |
| Female | 455(26.0%) |  | 0.71(0.51-0.98) | 0.035 |  | 0.71(0.51-0.97) | 0.034 |
| EBV (1 × 10^3^ copies/mL) | | |  |  |  | *P*≥0.05 | |
| <1 | 852(48.6%) | | 1(reference) | |  |  |  |
| <10 | 493(28.1%) | | 1.35(0.98-1.85) | 0.068 |  |  |  |
| ≥10 | 407(23.2%) | | 0.92(0.64-1.31) | 0.642 |  |  |  |
| Treatment |  |  |  |  |  |  |  |
| IMRT | 225(12.8%) | | 1(reference) | |  |  |  |
| CCRT | 635(36.2%) | | 0.94(0.57-1.55) | 0.799 |  |  |  |
| IC+CCRT | 892(50.9%) | | 0.76(0.46-1.27) | 0.299 |  |  |  |

**Abbreviation:** EBV, Epstein–Barr virus; WHO, World Health Organization; CCRT, concurrent chemoradiotherapy; IMRT, intensity-modulated radiation therapy; IC, induction chemotherapy

Note: HR and *P* value were calculated using multivariate Cox regression analysis.

**Table S3.** Percentage of skull base foramen invasion with different T categories (N=1752)

| Variables | Total | T1 | T2 | T3 | T4 |
| --- | --- | --- | --- | --- | --- |
| Foramen lacerum | |  |  |  |  |
| None | 1269(72.4%) | 450(99.8%) | 213(100%) | 494(72.9%) | 112(27.3%) |
| Yes | 483(27.6%) | 1(0.2%) | 0(0%) | 184(27.1%) | 298(72.7%) |
| Pterygopalatine fossa |  |  |  |  |  |
| None | 1374(78.4%) | 451(100%) | 210(98.6%) | 549(81%) | 164(40%) |
| Yes | 378(21.6%) | 0(0%) | 3(1.4%) | 129(19%) | 246(60%) |
| Foramen ovale |  |  |  |  |  |
| None | 1439(82.1%) | 451(100%) | 212(99.5%) | 612(90.3%) | 164(40%) |
| Yes | 313(17.9%) | 0(0%) | 1(0.5%) | 66(9.7%) | 246(60%) |
| Hypoglossal canal |  |  |  |  |  |
| None | 1593(90.9%) | 451(100%) | 213(100%) | 648(95.6%) | 281(68.5%) |
| Yes | 159(9.1%) | 0(0%) | 0(0%) | 30(4.4%) | 129(31.5%) |
| Jugular foramen |  |  |  |  |  |
| None | 1684(96.1%) | 451(100%) | 213(100%) | 664(97.9%) | 356(86.8%) |
| Yes | 68(3.9%) | 0(0%) | 0(0%) | 14(2.1%) | 54(13.2%) |
| Superior orbital fissure |  |  |  |  |  |
| None | 1740(99.3%) | 451(100%) | 213(100%) | 678(100%) | 398(97.1%) |
| Yes | 12(0.7%) | 0(0%) | 0(0%) | 0(0%) | 12(2.9%) |
| Inferior orbital fissure |  |  |  |  |  |
| None | 1668(95.2%) | 451(100%) | 213(100%) | 672(99.1%) | 332(81%) |
| Yes | 84(4.8%) | 0(0%) | 0(0%) | 6(0.9%) | 78(19%) |
| SBFI 2 classification |  |  |  |  |  |
| Non-severe | 1352(77.2%) | 451(100%) | 212(99.5%) | 586(86.4%) | 103(25.1%) |
| Severe | 400(22.8%) | 0(0%) | 1(0.5%) | 92(13.6%) | 307(74.9%) |
| SBFI 3 classification |  |  |  |  |  |
| None | 1070(61.1%) | 450(99.8%) | 209(98.1%) | 384(56.6%) | 27(6.6%) |
| Slight | 282(16.1%) | 1(0.2%) | 3(1.4%) | 202(29.8%) | 76(18.5%) |
| Severe | 400(22.8%) | 0(0%) | 1(0.5%) | 92(13.6%) | 307(74.9%) |

**Abbreviations:** SBFI, skull base foramen invasion; none, patients without SBFI; slight SBFI, patients with invasion foramen lacerum and/or pterygopalatine fossa invasion only; non-severe SBFI, patients without skull base foramina invasion and patients with only lacerum and/or pterygopalatine fossa invasion; severe SBFI, patients with other SBFIs.

**Table S4.** Multivariable analysis of skull base foramen invasion for total and T3 NPC patients

| SBFI |  | total (n=1752) |  |  |  | T3 (n=678) |  |
| --- | --- | --- | --- | --- | --- | --- | --- |
|  | N | OS HR(CI) | *P* value |  | N | OS HR(CI) | *P* value |
| Foramen lacerum | 483 | 1.33(0.97-1.83) | 0.076 |  | 184 | 1.41(0.92-2.16) | 0.112 |
| Pterygopalatine fossa | 378 | 1.12(0.82-1.54) | 0.463 |  | 129 | 1.16(0.72-1.88) | 0.543 |
| Foramen ovale | 313 | 1.09(0.77-1.55) | 0.633 |  | 66 | 1.75(0.98-3.12) | 0.059 |
| Hypoglossal canal | 159 | 1.21(0.83-1.76) | 0.314 |  | 30 | 1.42(0.65-3.12) | 0.379 |
| Jugular foramen | 68 | 2.28(1.49-3.48) | <0.001 |  | 14 | 2.03(0.81-5.11) | 0.131 |
| Superior orbital fissure | 12 | 2.05(0.83-5.07) | 0.119 |  | 0 | NA | NA |
| Inferior orbital fissure | 84 | 0.96(0.58-1.59) | 0.869 |  | 6 | 1.55(0.37-6.46) | 0.548 |
| Rather than foramen lacerum and/or pterygopalatine fossa | 400 | 1.45(1.01-2.08) | 0.047 |  | 92 | 1.94(1.19-3.18) | 0.008 |
| SBFI classification |  |  |  |  |  |  |  |
| None |  | 1(reference) |  |  |  | 1(reference) |  |
| Slight | 282 | 1.34(0.90-2.01) | 0.151 |  | 202 | 1.18(0.74-1.9) | 0.487 |
| Severe | 400 | 1.71(1.11-2.64) | 0.015 |  | 92 | 2.07(1.22-3.49) | 0.007 |

**Abbreviations:** NPC, nasopharyngeal carcinoma; HR, hazard ratio; CI, confidence interval; NA, not available; SBFI, skull base foramina invasion, none SBFI, patients without SBFI, slight SBFI, patients with invasion of foramen lacerum and/or pterygopalatine fossa only; severe SBI, patients with other SBFIs.

*: According to the Table 1, the following variables were included as the confounding factors: age, sex, T category and N category.

Note: *P* value was obtained from multivariate Cox regression analysis.

| variables | Severe SBFI | |  |  | non_severe SBFI | |  |
| --- | --- | --- | --- | --- | --- | --- | --- |
|  | non_IC | IC | *P* value |  | non_IC | IC | *P* value |
|  | (n=110) | (n=110) |  |  | (n=299) | (n=299) |  |
| Age  Median (IQR) | 48(40~58) | 46(37~55) | 0.059 |  | 47(40~56) | 44(38~52) | 0.009 |
| Sex |  |  | 1.000 |  |  |  | 1.000 |
| Male | 80(72.7%) | 80(72.7%) |  |  | 229(76.6%) | 229(76.6%) |  |
| Female | 30(27.3%) | 30(27.3%) |  |  | 70(23.4%) | 70(23.4%) |  |
| Histologic type |  |  | 0.620 |  |  |  | 0.841 |
| WHO type 1/2 | 1(0.9%) | 3(2.7%) |  |  | 14(4.7%) | 12(4.0%) |  |
| WHO type 3 | 109(99.1%) | 107(97.3%) |  |  | 285(95.3%) | 287(96.0%) |  |
| EBV (1 × 103 copies/mL) |  |  | 0.105 |  |  |  | <0.001 |
| <1 | 39(35.5%) | 26(23.6%) |  |  | 166(55.5%) | 99(33.1%) |  |
| <10 | 34(30.9%) | 34(30.9%) |  |  | 75(25.1%) | 97(32.4%) |  |
| ≥10 | 37(33.6%) | 50(45.5%) |  |  | 58(19.4%) | 103(34.4%) |  |
| T category |  |  | 1.000 |  |  |  | 1.000 |
| T1 | - | - |  |  | 19(6.4%) | 19(6.4%) |  |
| T2 | - | - |  |  | 18(6.0%) | 18(6.0%) |  |
| T3 | 28(25.5%) | 28(25.5%) |  |  | 235(78.6%) | 235(78.6%) |  |
| T4 | 82(74.5%) | 82(74.5%) |  |  | 27(9.0%) | 27(9.0%) |  |
| N category |  |  | 1.000 |  |  |  | 1.000 |
| N0 | 21(19.1%) | 21(19.1%) |  |  | 29(9.7%) | 29(9.7%) |  |
| N1 | 65(59.1%) | 65(59.1%) |  |  | 180(60.2%) | 180(60.2%) |  |
| N2 | 23(20.9%) | 23(20.9%) |  |  | 72(24.1%) | 72(24.1%) |  |
| N3 | 1(0.9%) | 1(0.9%) |  |  | 18(6%) | 18(6%) |  |
| Stage |  |  | 1.000 |  |  |  | 1.000 |
| III | 28(25.5%) | 28(25.5%) |  |  | 255(85.3%) | 255(85.3%) |  |
| IVa | 82(74.5%) | 82(74.5%) |  |  | 44(14.7%) | 44(14.7%) |  |
| GTV (cm^3^)  Median (IQR) | 66.7(41.5~89.3) | 61(38~79.9) | 0.179 |  | 31.2(22.4~47) | 33.8(21.7~47.6) | 0.922 |
| Lymph nodal number  Median (IQR) | 2(1~4) | 2(1~4) | 0.814 |  | 3(1~5) | 4(2~7) | 0.013 |

**Table S5.** Basic characteristics of SBFI subclassification treated with or without induction chemotherapy in 1:1 random matched-pair analysis.

**Abbreviation:** SBFI, skull base foramina invasion; non_severe SBFI, patients without skull base foramina invasion and patients with only lacerum and/or pterygopalatine fossa invasion; severe SBFI, patients with other SBFIs; IQR: interquartile range; IC, induction chemotherapy; EBV, Epstein–Barr virus; WHO, World Health Organization; GTV, gross tumor volume.

Note 1: Each pair was matched by confounding variables of age, sex, T category and N category among patients with III~IVa stage nasopharyngeal carcinoma.

Note 2: *P* values were calculated using chi-square analysis or Fisher’s exact test for categorical variables, and Student’s t-test or Mann-Whitney U-test for continuous variables.

Note 3: Continuous variables were described as median (interquartile range).

**Table S6.** C-index of the proposed T staging system in NPC patients

|  | OS | C-index(95%CI) | |
| --- | --- | --- | --- |
|  |  | Train (n=1320) | Test (n=432) |
| T | Proposed T | 0.675(0.638-0.712) | 0.677(0.620-0.735) |
|  | 8th edition T | 0.658(0.620-0.695) | 0.642(0.584-0.701) |
|  | *P* value^†^ | 0.850 | 0.455 |
|  |  |  |  |
| T+cf | Proposed T | 0.746(0.710-0.782) | 0.732(0.678-0.785) |
|  | 8th edition T | 0.742(0.705-0.778) | 0.717(0.662-0.771) |
|  | *P* value^†^ | 0.216 | 0.240 |

**Abbreviations:** C-index, Harrell concordance index; CI, confidence interval; OS, overall survival; T, T staging system.

cf, confounding factors, included: T category, N category, Age, and Sex.

*P* value†：U-statistics test was used to compare two C-index, mainly using the rcorrp.cens function in the Hmisc package of R


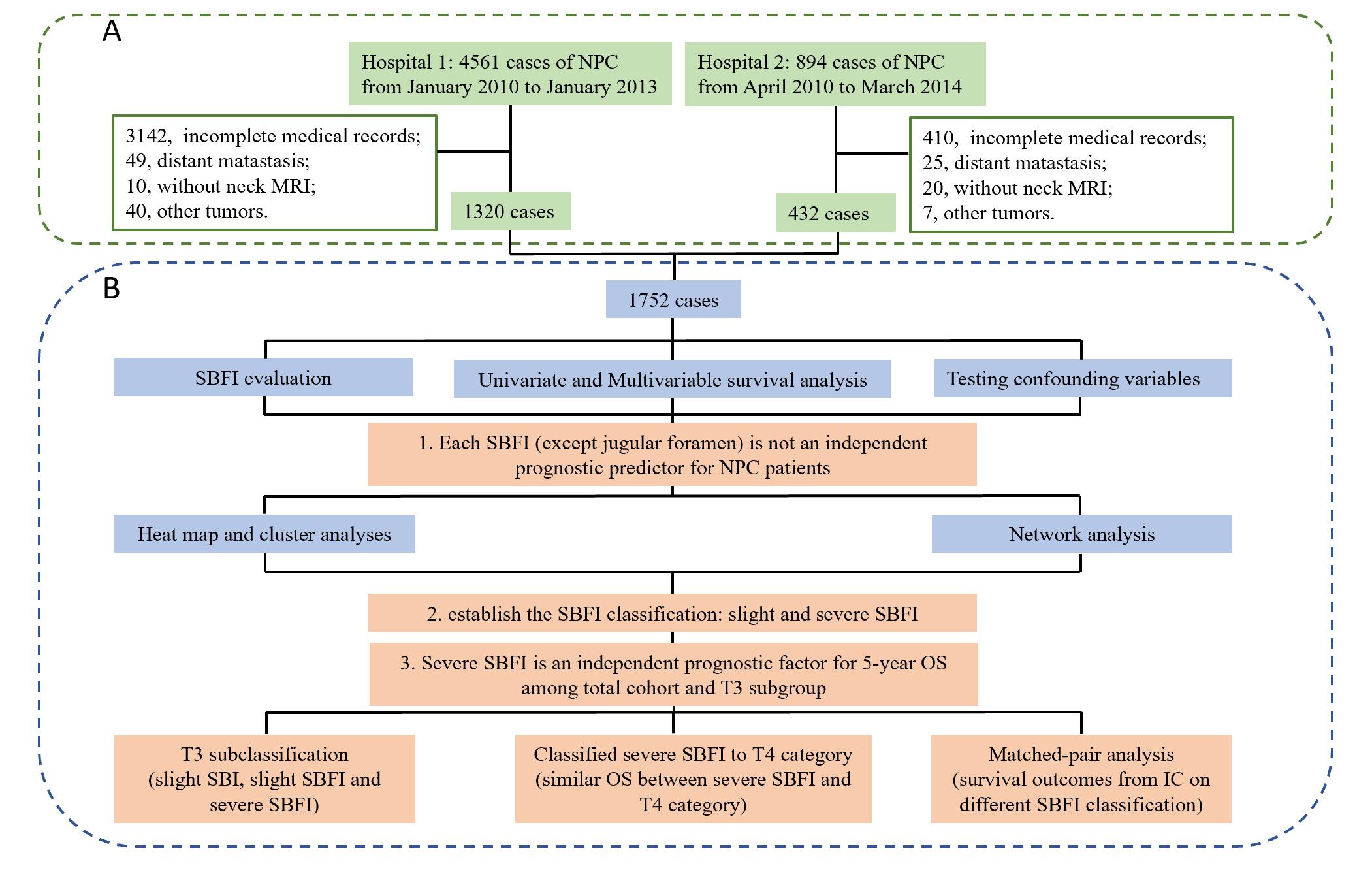
**Figure S1 Flowchart of the study: patient recruitment (A) and procedure of analysis (B).**

**Abbreviations:** NPC, nasopharyngeal carcinoma; SBFI, skull base foramen invasion; OS, overall survival; IC, induction chemotherapy

**Figure S2. Independent validation of prognostic value of severe SBFI in T3 patients with nasopharyngeal carcinoma.**


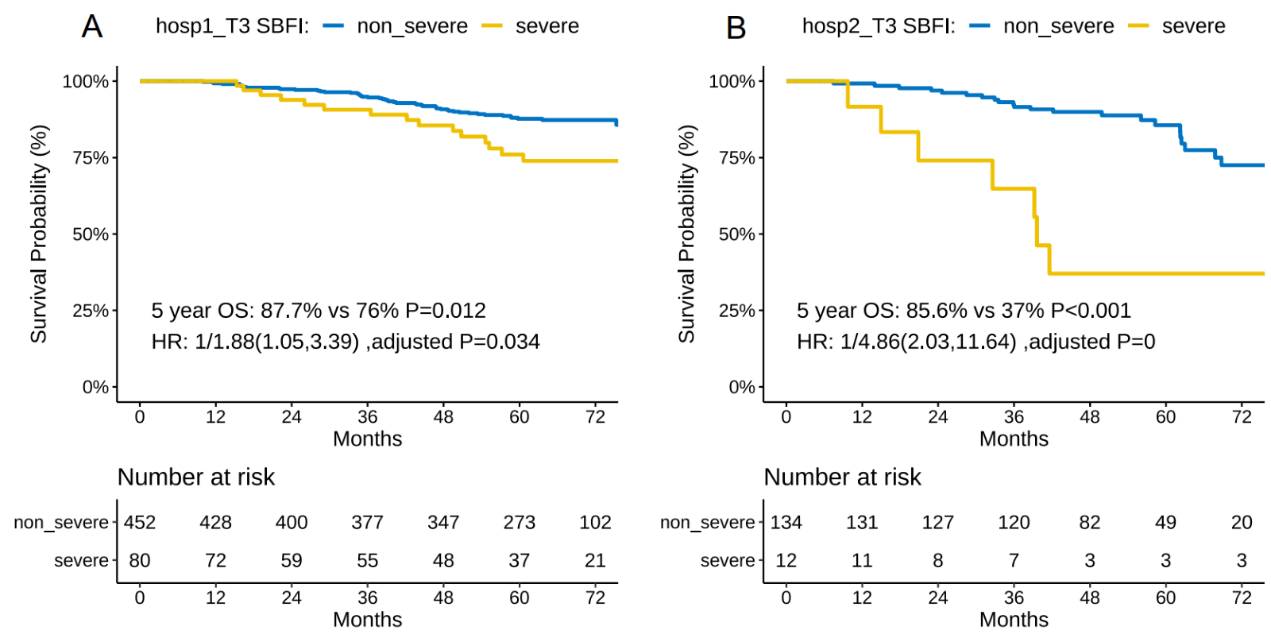


Severe SBFI is an independent adverse prognostic factor for OS among T3 patients with nasopharyngeal carcinoma (NPC) in both hospitals (all *P*<0.05).

**Abbreviations:** HR, hazard ratio; OS, overall survival; non_severe SBFI, patients without skull base foramina invasion and patients with only lacerum and/or pterygopalatine fossa invasion; severe SBFI, patients with other SBFIs.

**Note.** *P* value was calculated by log-rank test; HR and adjusted *P* value were calculated by multivariable Cox regression with confounding factors.

**Figure S3: The 5-year OS for T staging according to the 8th edition AJCC system for hospital 1 and 2.**

**
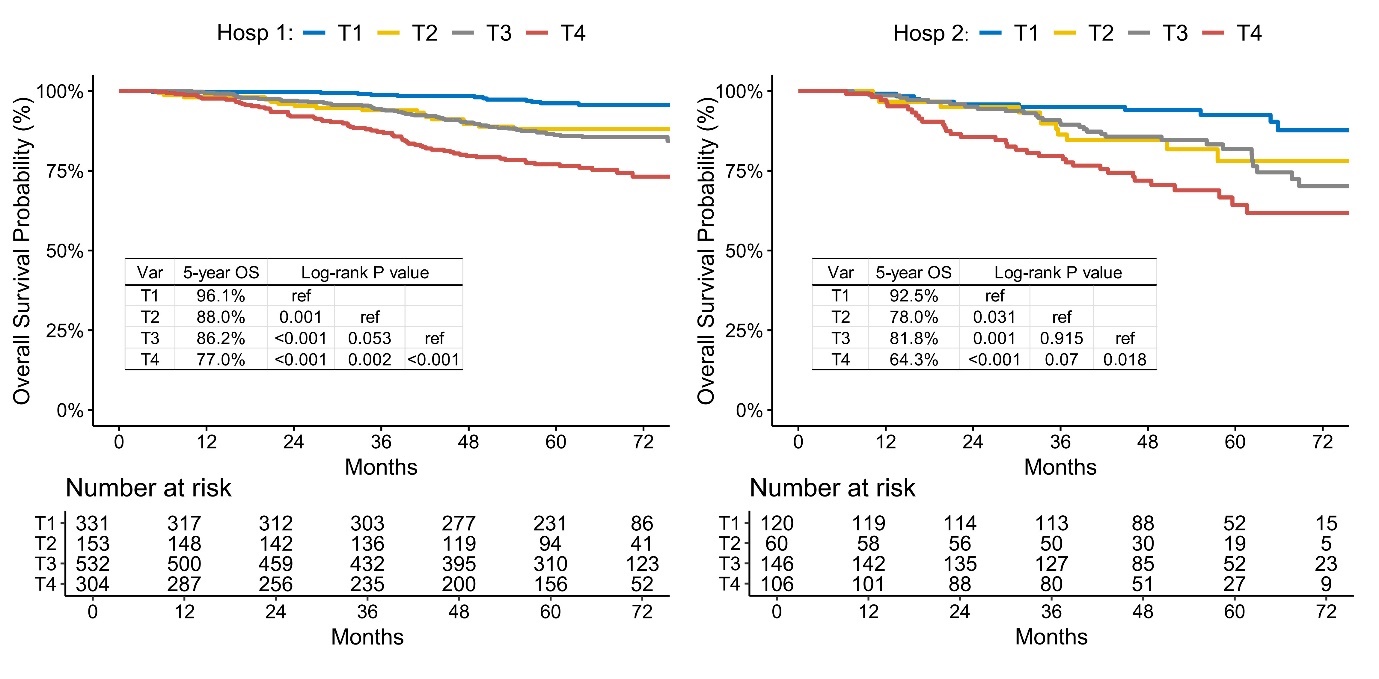
**

The 5-year OS for T2 and T3 categories almost overlapped in both hospitals.

**Abbreviations:** AJCC, American Joint Committee on Cancer; HR, hazard ratio; OS, overall survival.

**Note.** *P* value was calculated by log-rank test.

**Figure S4. Survival outcomes for 5-year OS among SBFI subclassification in T3 and T4 patients with nasopharyngeal carcinoma.**


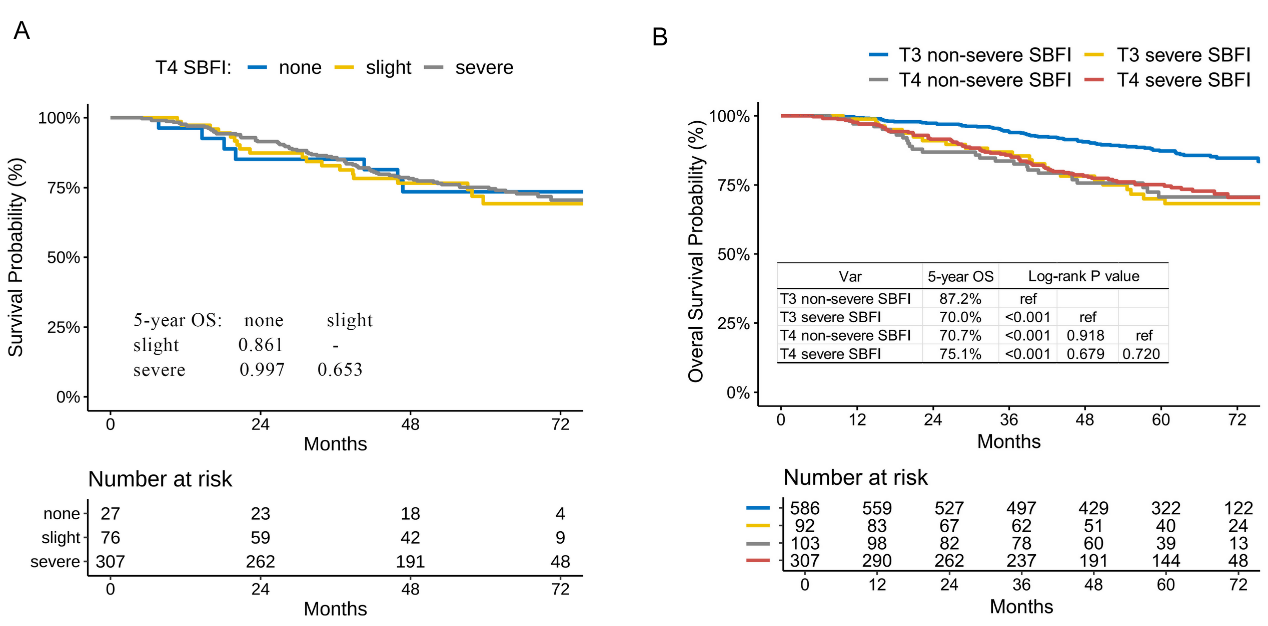


The 5-year OS among T4 patients with different SBFI subclassification were overlapped (A). T3 patients with non_severe SBFI exhibited significantly better OS compared to the rest patients within the group(*P*<0.05) (B).

**Abbreviations**: HR, hazard ratio; OS, overall survival; non_severe SBFI, patients without skull base foramina invasion and patients with only lacerum and/or pterygopalatine fossa invasion; severe SBFI, patients with other SBFIs.

**Note.** *P* value was calculated by log-rank test.

**Figure S5. Survival outcomes for PFS among SBFI subclassification in T3 and T4 patients with nasopharyngeal carcinoma.**


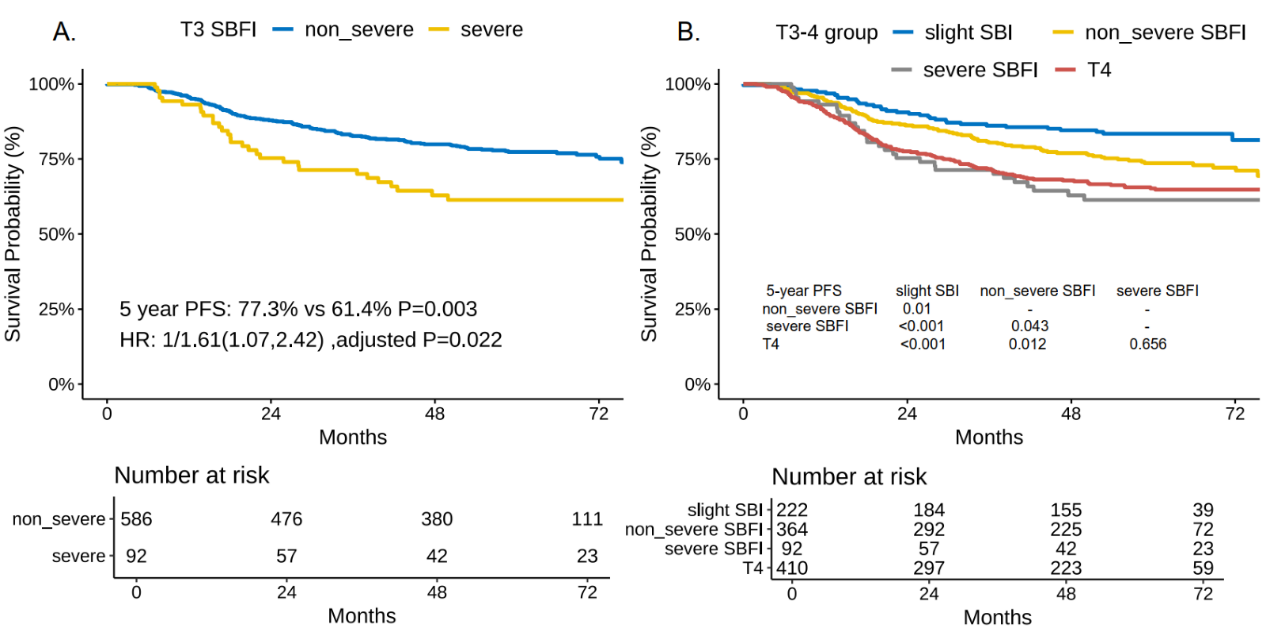


The PFS results underscore the adverse prognostic impact of severe SBFI (P<0.05) (A), PFS among T3 patients with severe SBFI overlapping those of the T4 group (B).

**Abbreviations:** HR, hazard ratio; CI, confidence interval; PFS, progression-free survival; SBFI, skull base foramen invasion; non_severe SBFI, patients without skull base foramina invasion and patients with only lacerum and/or pterygopalatine fossa invasion; severe SBFI, patients with other SBFIs; slight SBI, T3-stage patients who skull base invasion only involved the pterygoid process and/or the base of sphenoid bone.

**Note.** *P* value was calculated by log-rank test; HR and adjusted P value were calculated by multivariable Cox regression with confounding factors.
